# Greater Phage Genotypic Diversity Constrains Arms-Race Coevolution

**DOI:** 10.3389/fcimb.2022.834406

**Published:** 2022-03-04

**Authors:** Meaghan Castledine, Pawel Sierocinski, Mhairi Inglis, Suzanne Kay, Alex Hayward, Angus Buckling, Daniel Padfield

**Affiliations:** College of Life and Environmental Sciences, Environment and Sustainability Institute, University of Exeter, Cornwall, United Kingdom

**Keywords:** bacteriophage, *Pseudomonas fluorescens*, genetic diversity, coevolution, resistance, infectivity, experimental evolution, arms race

## Abstract

Antagonistic coevolution between hosts and parasites, the reciprocal evolution of host resistance and parasite infectivity, has important implications in ecology and evolution. The dynamics of coevolution—notably whether host or parasite has an evolutionary advantage—is greatly affected by the relative amount of genetic variation in host resistance and parasite infectivity traits. While studies have manipulated genetic diversity during coevolution, such as by increasing mutation rates, it is unclear how starting genetic diversity affects host–parasite coevolution. Here, we (co)evolved the bacterium *Pseudomonas fluorescens* SBW25 and two bacteriophage genotypes of its lytic phage SBW25ɸ2 in isolation (one phage genotype) and together (two phage genotypes). Bacterial populations rapidly evolved phage resistance, and phage reciprocally increased their infectivity in response. When phage populations were evolved with bacteria in isolation, bacterial resistance and phage infectivity increased through time, indicative of arms-race coevolution. In contrast, when both phage genotypes were together, bacteria did not increase their resistance in response to increasing phage infectivity. This was likely due to bacteria being unable to evolve resistance to both phage *via* the same mutations. These results suggest that increasing initial parasite genotypic diversity can give parasites an evolutionary advantage that arrests long-term coevolution. This study has important implications for the applied use of phage in phage therapy and in understanding host–parasite dynamics in broader ecological and evolutionary theory.

## Introduction

Antagonistic coevolution between hosts and parasites, the reciprocal evolution of host resistance and parasite infectivity, has important implications for a range of ecological and evolutionary processes (Brockhurst and Koskella, 2013; Jephcott et al., 2016). Of key importance is which species has the evolutionary advantage in the interaction and, hence, is better adapted (Brockhurst and Koskella, 2013). The rate of adaptation will be positively affected by the relative amount of genetic variation underpinning host defense and parasite infectivity traits (Dybdahl et al., 2014). Hence, mechanisms that increase genetic variation, such as sexual reproduction, increased mutation rates, and increased immigration rates at relevant loci in one partner relative to another, can be beneficial (Morgan et al., 2005; Greischar and Koskella, 2007; Morran et al., 2011; Savolainen et al., 2013; Lynch et al., 2016; Papkou et al., 2016). Most studies to date have investigated conditions where differences in genetic variation between host and parasite are manipulated throughout coevolution (Morgan et al., 2005; Morran et al., 2011; Brockhurst and Koskella, 2013; Savolainen et al., 2013; Jephcott et al., 2016; Zhang and Buckling, 2016). What is less clear is the importance of differences in relative standing genetic variation, which will inevitably vary considerably between different pairs of host–parasite populations of the same species (King and Lively, 2012; Forsman and Wennersten, 2016). Specifically, is any advantage to host or parasite short lived or does initial diversity have implications for long-term coevolutionary dynamics?

We investigated this question using coevolving bacteria and phage (*Pseudomonas fluorescens* SBW25 and SBW25ɸ2) in experimental microcosms (Buckling and Rainey, 2002). We use bacteria and phage because of their propensity to rapidly coevolve in the laboratory (Buckling and Rainey, 2002) and because of the potential applied relevance of enhancing phage infectivity when phages are used therapeutically (Abedon, 2019; Kwiatek et al., 2020). Phages in this system usually have a disadvantage during coevolution as it is often easier for bacteria to lose receptors that phages bind to than it is for phages to adapt to new or modified receptor sites (Morgan et al., 2010; van Houte et al., 2016). Hence, we focused on investigating the consequences of increased initial phage genetic diversity on the outcome of coevolution. Our design was very simple: we coevolved initially isogenic populations of bacteria against two very closely related phage genotypes, either singly or together. We then measured levels of evolved resistance and infectivity of bacteria and phage, respectively, through time. We additionally carried out time-shift assays (i.e., measured infectivity of phage to bacteria within and across time-points) to determine coevolutionary dynamics. Specifically, we were interested in the rate of coevolution and the extent to which coevolution followed an escalatory arms race, with bacteria retaining resistance to previously encountered phage genotypes, and phages retaining resistance to previously encountered host genotypes.

## Methods

### Bacteria and Phage Strains

Two phage strains were used in this study: the wild-type lytic bacteriophage SBW25ɸ2 (GenBank accession number FN594518) (termed P1 in this study) and the other an almost identical isolate (P2). This new isolate was discovered when plating out wastewater onto SBW25 in an attempt to isolate novel phages, but the genetic similarity (see below) between the new isolate and SBW25ɸ2 suggests that it was most likely a laboratory contaminant from a previous coevolution experiment rather than an independently isolated phage. An important factor that will determine whether increased genetic variation will be advantageous to the phages is whether resistance to one phage confers resistance to the other (cross-resistance) (Wright et al., 2019). Increased phage diversity is unlikely to be beneficial if there was extensive cross-resistance. Cross-resistance was tested by growing *P. fluorescens* SBW25 overnight at 28°C, shaking, with each phage in monoculture (6 replicates per phage) in 6 ml King’s medium B (KB) before plating onto KB agar (King et al., 1954). Previous work has shown that high levels of resistance evolve in SBW25 over this timeframe under these conditions (Buckling and Rainey, 2002; Padfield et al., 2020). Plates were incubated for 2 days at 28°C, after which colonies were picked from each monoculture treatment replicate. Each colony was tested for resistance to the phage it was cultured with using phage streak assays (Buckling and Rainey, 2002). 12 phage-resistant colonies were isolated from each replicate and tested for resistance to the opposite phage using streak assays. Plates were incubated for 24 h, and bacterial clones were scored as resistant (0) or susceptible (1) to each phage.

### Experimental Evolution

Evolution lines of *P. fluorescens* SBW25 and two genotypes of SBW25ɸ2 were established with two monoculture (P1 or P2) lines and one polyculture (P1 and P2) line with 12 replicates per line. To monoculture lines, 10 µl of phage suspension was added while in the polyculture line, 5 µl of each phage was added (10 µl total). Evolution lines were cultured unshaken in 5 ml KB at 28°C in glass microcosms with loosened plastic lids. Serial 100-fold dilutions (50 µl into 5 ml) took place every 48 h for 6 total transfers (total of 12 days). At each transfer, 150 µl of culture was frozen at -80°C with 75 µl 60% glycerol and phage was extracted *via* chloroform extraction (Sechaud and Kellenberger, 1956). 900 µl of culture and 100 µl of chloroform were vortexed then centrifuged at 13,000 g for 5 min. The supernatant was removed and refrigerated at 4°C.

### Measuring Coevolution

A time-shift assay was used to estimate the coevolution and type of dynamics (Buckling and Rainey, 2002). Coevolution was estimated over 3 time-points of bacteria and phage evolution: days 4, 8, and 12. From each time-point and treatment replicate, 20 µl of frozen culture was inoculated into 180 µl KB medium in 96-well plates and grown for 24 h at 28°C. Overnight cultures were plated onto KB agar as previously described, and 12 bacterial colonies were picked from each time-point and treatment replicate. Bacterial colonies were inoculated into 150 µl KB and frozen as above. Changes in bacterial resistance/phage infectivity were estimated through spot assays (Kutter, 2009). Phage extractions from each time-point were spotted onto lawns of individual bacterial clones from all time-points within corresponding treatment replicates. Plates were incubated for 24 h, and bacterial clones were scored as resistant (0) or susceptible (1) to each phage extract.

### Sequencing

The mutant strain of SBW25ɸ2(P2) was sequenced to assess how different it was to the ancestral SBW25ɸ2. Briefly, the phage was grown with SBW25 (as described above) to high titers (0.6 × 10^6^ plaque-forming units/ml) before chloroform extraction (4 ml culture; 400 µl chloroform). The supernatant (4 ml) was treated with DNase I (20 U/ml for ~20 min), after which a 100-kDa Amicon Ultra-0.5 centrifugal filter unit was used to remove the DNase-I enzyme and concentrate phage for DNA extraction. Phage lysis and DNA extraction were performed using the Thermo Fisher GeneJET Genomic DNA Purification Kit following the manufacturer’s instructions for gram-negative cells. PacBio RS II using C4/P6 chemistry was used to generate long reads at the Centre for Genomic Research (Liverpool, UK). PacBio reads were processed to give 170 individual PacBio-corrected reads (minimum size = 517 bp, maximum size = 28,383 bp, median = 4,424 bp). These corrected reads were mapped to the reference SBW25ɸ2 genome (NCBI genome accession number: GCA_000886135.1) using *minimap2* (v2.23-r1111) with the options “*-ax map-pb*” (100% of reads mapped) (Li, 2018). Variants were called using *freebayes* (v1.3.5) (Garrison and Marth, 2012) with ploidy set to 1 (*-p 1*), and *vcffilter* from *vcflib* was used to only keep variants with a quality score of >20 (Garrison, 2012). The ancestral (laboratory stock; P1) SBW25ɸ2 was resequenced separately on an Illumina HiSeq 4000 to produce 2 × 150-bp paired-end sequencing to check whether any mutations had occurred during stock maintenance. Reads were trimmed for the presence of Illumina adapter sequences, for quality scores below 10, and for paired-end reads where one read was shorter than 20 bp by TrimGalore (v0.6.7) (Krueger et al., 2021). Reads were mapped to the reference genome and variants called as above (options for minimap2 “*-ax sr*”).

### Statistical Analyses

All data were analysed using R (v. 4.0) in RStudio (Team, 2013), and all plots were made using the package “*ggplot2*” (Wickham, 2016). Changes in bacterial resistance/phage infectivity were analysed using a generalised linear mixed effects model with a binomial error structure. First, we analysed whether phage infectivity to contemporary bacteria varied over time within each treatment. For this model, the time-shift assays were subset so we only retained assays where phage and bacteria were from the same time-point. The proportion of susceptible bacterial clones was analysed against interacting fixed effects of time-point and treatment (P1, P2, or both phage combined). Next, we assessed whether bacteria and phage coevolved in a model where the proportion of susceptible bacterial clones was analysed against interacting fixed effects of bacterial time-point, phage time-point, and treatment (P1, P2 or P1 and P2). A random effect of treatment replicate was included to account for which evolution line replicate colonies were isolated from, and an observation level was included due to model overdispersion (Harrison, 2014). If bacteria and phage evolve by an ARD, we would expect the phage infectivity to increase with phage time-point while decreasing with bacterial time-point, whereas, if FSD results, phage infectivity should show a non-linear relationship with phage and bacteria time-points, with bacteria being more resistant to phage originating from their contemporary time-point. Models were fitted using the package “*lme4*” (Bates et al., 2014). Model simplification was conducted using likelihood ratio tests, and Tukey’s *post-hoc* multiple-comparison tests were performed on the most parsimonious model using the R package “*emmeans*” (Lenth, 2018).

## Results

### Phage Strains

The two phage strains were similar to the reference SBW25ɸ2. As expected, the ancestral-type phage (P1) differed by few, only three SNPs to the reference SBW25ɸ2, with one of these SNPs being in the tail-fibre gene which is known to be important for phage infectivity (Scanlan et al., 2011). The new isolate (P2) shared the SNP in the tail-fibre gene but also differed in 5 other genetic variants (SNPs and indels), with two also being in the tail-fibre gene (**Table 1**). We looked at whether evolutionary differences resulted in phenotypic differences by culturing *P. fluorescens* with each phage in monoculture for 24 h and testing whether cross-resistance emerged. Bacterial colonies (n = 12) resistant to P2 remained susceptible to P1. In contrast, 51.4 ± 0.062% (SE; standard error) of bacterial clones resistant to P1 were resistant to P2 showing moderate levels of cross-resistance.

**Table 1:** Genetic differences between Phage 1 (P1) and Phage 2 (P2).

Phage variant	Genome position	Reference sequence	Alternative variant	Variant in a putative gene	Variant in a tail fiber gene
Phage 1	**37,265**	**A**	**G**	**Yes**	**Yes**
	42,395	C	T	Yes	No
	42,619	C	G	No	
Phage 2	345	ACCTA	ACTA	No	
	1,186	ACCAAGGCCAAGGT	ACCAAGGT	Yes	No
	3,920	G	A	Yes	No
	5,220	GGGTGTGGGA	GGGGGA	Yes	No
	35,880	A	G	Yes	Yes
	35,979	C	T	Yes	Yes
	**37,265**	**A**	**G**	**Yes**	**Yes**

Shared genetic variants are in bold.

### Phage Infectivity to Co-Occurring Bacteria

**Figure 1:**
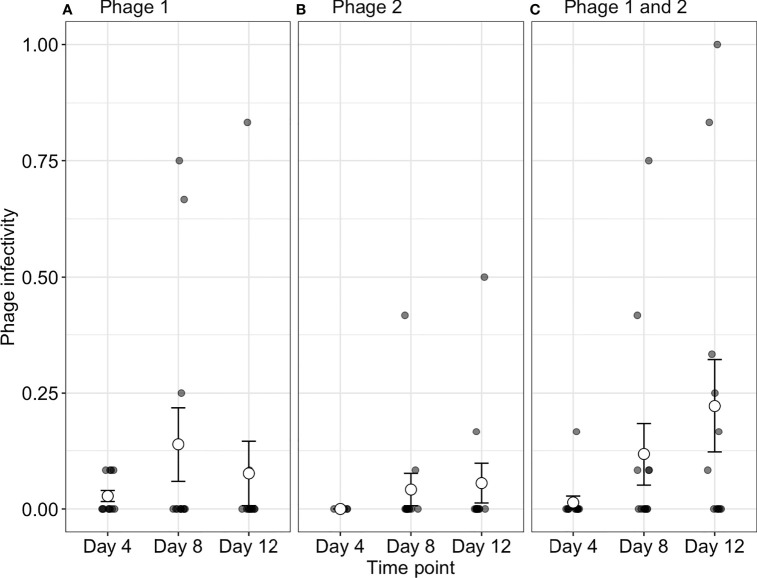
Phage infectivity toward their contemporary bacterial populations. Infectivity is broadly consistent over time when phages are in monoculture **(A, B)**. In contrast, when phages are together in polyculture **(C)**, infectivity increases through time (i.e., the proportion of phage susceptible bacteria increases). Small points indicate the proportion of bacterial clones infected within a single treatment replicate. Large points represent the means of phage infectivity for phage isolated and tested against bacteria from different time-points. The bars show the ± SEs. Phage 1, P1; Phage 2, P2.

To determine if increased starting phage diversity provides an advantage during coevolution, we determined the infectivity of phages to their contemporary bacteria. Although bacterial populations were initially sensitive to both phage genotypes, we observed high levels of phage resistance with 88.4 ± 2.203%, 86.7 ± 2.697%, and 91.3 ± 2.090% of all bacterial clones being phage resistant (averaged over all time-points) across P1, P2, and phage-together treatments, respectively (**Figure 1**). While resistance evolved to high levels across treatments, we observed changes in contemporary resistance through time. Phage infectivity to contemporary bacteria was affected by a significant interaction of treatment and time-point (ANOVA comparing models with and without treatment × time interaction: $\chi_{4}^{2}$ = 16.33, p = 0.003). In monoculture treatments, phage infectivity remained broadly consistent over time with no significant differences in P2 (Tukey HSD: all p-values > 0.05; **Table S1**) and a significant increase in infectivity only observed in P1 between days 4 (infection probability = 0.003, 95% CI = 0.0004–0.018) and 8 (infection probability = 0.03, 95% CI = 0.006–0.136; Tukey HSD: p = 0.001; **Figure 1** and **Table S1**). In contrast, when both phages were together, the proportion of bacteria susceptible to their contemporary phage increased through days 4, 8, and 12 (Tukey HSD: all p-values < 0.05; **Table S1**). In other words, contemporary phage resistance decreased over time (**Figure 1** and **Table S1, S2**).

### Coevolutionary Dynamics

**Figure 2:**
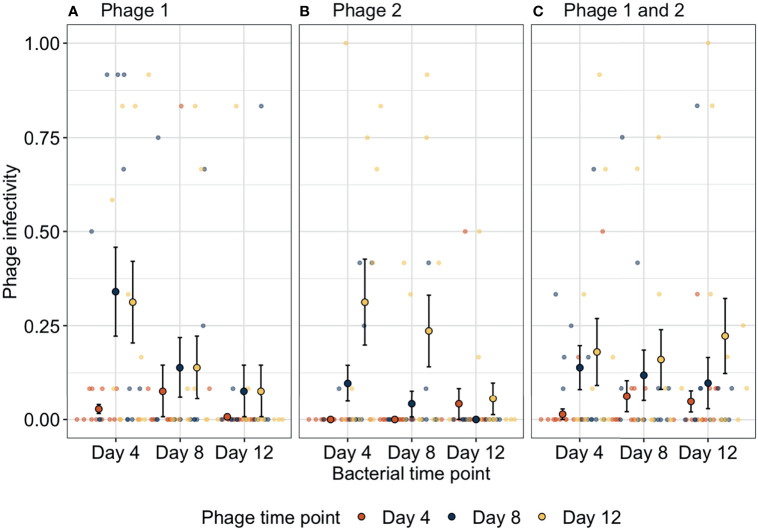
Changes in phage infectivity to bacteria isolated from three different time-points over bacteria-phage coevolution. In the presence of **(A)** strain one or **(B)** strain two, bacteria coevolved with phage with increasing bacterial resistance and phage infectivity through time (arms-race coevolution). In contrast, when **(C)** both phage genotypes were together, phage infectivity increased through time; however, bacteria did not reciprocally increase in resistance indicating a lack of coevolution. Small points indicate the proportion of bacterial clones infected within a single treatment replicate. Large points represent the means of phage infectivity for phage isolated and tested against bacteria from different time-points. The bars show the ± SEs. Phage 1, P1; Phage 2, P2.

We determined how bacteria and phage were coevolving by exposing bacterial populations from different time-points to phage from three time-points. Bacteria-phage populations can coevolve *via* arms-race dynamics (ARD) in which bacterial resistance and phage infectivity increase through time (Buckling and Rainey, 2002). In contrast, phage can specialise on specific host genotypes resulting in selection of rare host genotypes that are resistant to the contemporary dominant phage population; this results in fluctuating selection dynamics (FSD) as bacteria and phage populations have a negative frequency-dependent selection (Gomez and Buckling, 2011). Here, phage infectivity against bacteria from any given time-point significantly increased through time (ANOVA between models containing phage time-point versus a model without: $\chi_{2}^{2}$ = 26.3, p < 0.001). Across treatments, infectivity at days 8 (infection probability = 0.004, 95% CI = 0.00127–0.01465) and 12 (infection probability = 0.015, 95% CI = 0.00481–0.04407) was significantly greater than that for phage from day 4 (infection probability = 0.0005, 95% CI = 0.00012–0.00211; Tukey HSD: p = 0.0016 and p < 0.0001, respectively) while phage infectivity was non-significantly different between days 8 and 12 (Tukey HSD: p = 0.0625, **Figure 2**). These patterns of increasing phage infectivity were consistent regardless of phage treatment.

Furthermore, bacteria showed increases in phage resistance against phage isolated from given time-points, but this pattern was not found in all treatments (ANOVA between models containing bacteria time-point phage treatment interaction versus a model without: $\chi_{4}^{2}=12.34$ , p = 0.015). In treatment P1, phage susceptibility decreased significantly between days 4 (susceptibility probability = 0.032, 95% CI = 0.006–0.144) and 8 (susceptibility probability = 0.003, 95% CI = 0.0004–0.02) and levelled off between days 8 and 12 (susceptibility probability = 0.0005, 95% CI = 0.00005–0.004). Similarly, in treatment P2 bacterial susceptibility decreased through time but decreases were slower as only bacteria from day 12 (susceptibility probability = 0.0003, 95% CI = 0.00003–0.003) were significantly less susceptible than bacteria from day 4 (susceptibility probability = 0.004, 95% CI = 0.0006–0.027). In contrast, when both genotypes were together in polyculture, no significant changes in bacterial susceptibility were found (Tukey HSD, all p-values > 0.05; **Table S3**). Overall, phage treatments P1 and P2 showed evidence of arms-race coevolutionary dynamics with reciprocal increases in phage infectivity and bacterial resistance through time as typified by this system under these culture conditions (Buckling and Rainey, 2002), whereas, when both phage genotypes were present, arms-race coevolution was constrained with initial increases in phage resistance between days 0 and 4, after which only phage evolved.

## Discussion

During this short coevolutionary experiment, bacteria evolved high levels of resistance to phages across all treatments. However, the dynamics of bacteria resistance and phage infectivity evolution differed between monoculture and when both phage genotypes were present, even though the genotypes only differed by 8 genetic variants (SNPs and indels). When each phage evolved in isolation with *P. fluorescens*, future phage isolates were more infective on bacterial populations from the past (e.g., day 4 bacterial populations vs. phage from days 8 and 12; **Figures 2A, B****)**. However, bacterial populations from the end of the experiment were almost entirely resistant to phage (e.g., day 12 bacteria vs. phage from days 4 and 8; **Figures 2A, B****)**. These dynamics of bacteria and phage are typical of ARD with increasing bacterial resistance and phage infectivity ranges evolving over time (Buckling and Rainey, 2002). In contrast, when both phage genotypes were together, bacteria evolved resistance and while phage evolved to increase infectivity, bacteria did not increase their resistance as we would expect under ARD. Interestingly, this led to declines in contemporary resistance over time. When both phage genotypes were together, bacteria rapidly evolved high levels of resistance but showed no evidence of further resistance evolution. In contrast, phage infectivity continued to increase which resulted in declines in bacterial resistance to contemporary phage.

Why bacteria were unable to increase their resistance in the presence of multiple genotypes is likely due to genetic constraints and/or costs of phage resistance (Gandon and Michalakis, 2002; Gandon et al., 2008; Ashby et al., 2014; Lopez Pascua et al., 2014; van Houte et al., 2016). *P. fluorescens* SWB25 typically evolves resistance to ɸ2 *via* surface modification which is associated with lower growth rates (Buckling et al., 2006; Zhang et al., 2021) and that may be increased if two phage genotypes select for greater receptor changes (van Houte et al., 2016). Changing receptor sites further may also be constrained if mutations increasing resistance to multiple phage genotypes simultaneously are less probable (Wright et al., 2019), or if resistance to one phage increases susceptibility to the other phage strain (Stoddard et al., 2007; Avrani et al., 2011; Castillo et al., 2014). Previous work suggests that the simultaneous exposure to diverse phages is likely to be important in constraining resistance evolution, with the constraints reduced if bacteria are exposed to different phages sequentially (Wright et al., 2019).

Increasing genetic diversity of phage could be beneficial in applied contexts (such as phage therapy) if arresting coevolution resulted in declines in phage-resistant bacterial populations (Torres-Barceló, 2018). Additionally, using multiple phages to limit resistance evolution is often the rationale of using phage cocktails which have been shown to be therapeutically more beneficial than single strains in treating bacterial infections (Hall et al., 2012; Alves et al., 2016; Chadha et al., 2016; Yen et al., 2017; Abedon et al., 2021). However, coevolution may be important in such contexts, and in ecological contexts where phages are hypothesised to be important in regulating community diversity, to eliminate the existing resistant populations (Hall et al., 2012; Jephcott et al., 2016; Jariah and Hakim, 2019; Wright et al., 2019; Broniewski et al., 2020). If phages can overcome resistance, they may maintain bacterial diversity in natural environments such as soils and seawater (Chevallereau et al., 2022), and in industrial settings such as cheese cultures (Erkus et al., 2013) and wastewater (Shapiro et al., 2010). In phage therapy, this challenge of phage resistance may be alleviated by the stressful conditions experienced by bacteria *in vivo* (e.g., challenged by the immune system, experience competition from microbiota, unfavourable pH) which may limit the potential for broad resistance evolution in bacteria by restricting replication rates (Everest, 2007; van Houte et al., 2016). Future research should aim to test the effects of phage genetic diversity in diverse environmental and ecological (e.g. community complexity) conditions.

In our model system, resistance primarily evolves *via* surface modification, but we might expect different results in bacterial populations that can evolve resistance *via* alternative mechanisms, such as CRISPR-Cas (clustered regularly interspaced short palindromic repeats; CRISPR-associated). In a recent study, increasing phage diversity decreased the probability of CRISPR resistance but may not give an advantage in coevolution as phage extinctions were the same irrespective of genetic diversity (Broniewski et al., 2020). Additionally, results may differ with temperate phages as bacteria can often carry multiple prophages on their genome, thereby conferring resistance to lytic infection from the same phage (Howard-Varona et al., 2017). Coevolutionary interactions with prophages have been shown to be short-lived with either bacteria becoming resistant by CRISPR or phage returning to a temperate state (from becoming lytic) (Cornuault et al., 2020).

To conclude, we find that increased phage diversity from one to two phage genotypes can constrain arms-race dynamics. Arms-race dynamics have been reported in a broad range of bacteria–phage pairs (Buckling and Rainey, 2002; Betts et al., 2014; Osada et al., 2017; Wandro et al., 2019) with diversity generated through *de novo* mutation following selection on isogenic populations (Brockhurst et al., 2004; Lopez Pascua et al., 2014). However, this is the first instance where initial phage diversity has been manipulated and has been shown to result in arms-race dynamics being constrained in the host bacterium, while only phages continue the “arms-race” of increasing infectivity. These results suggest that initial phage genetic diversity is important in determining the (co)evolutionary trajectories of bacteria–phage interactions. Understanding the effects of phage genetic diversity on bacterial evolution has implications in natural and applied contexts. More generally, while (co)evolution in many other (non-bacteria–virus) natural systems is expected to be slower due to longer generational times (Penczykowski et al., 2016), our results suggest that coevolution may be constrained where host populations have to adapt to multiple parasite genotypes. This is in line with a broader theory that complex ecosystems, which contain multiple and often conflicting selection pressures, can slow rates of adaptation by inducing trade-offs or requiring multiple evolutionary steps (e.g., co-occurring mutations) for host–parasite fitness peaks to be reached (Gandon and Michalakis, 2002; Penczykowski et al., 2016).

## Data Availability Statement

All phenotypic data and code used in the analysis is available on GitHub (https://github.com/padpadpadpad/Castledine_2022_frontiers). The raw sequencing files are archived on the European Nucelotide Archive (Experiment accession number: PRJEB50009).

## Author Contributions

Study conceived and designed by PS, MC, and AB. Experiments conducted and data collected by PS, SK, and MI. Data analysis conducted by MC, DP, and AH. All authors contributed to the article and approved the submitted version.

## Funding

This work was supported in part by grant MR/N0137941/1 awarded to MC for the GW4 BIOMED MRC DTP, awarded to the Universities of Bath, Bristol, Cardiff and Exeter from the Medical Research Council (MRC)/UKRI. AH is supported by a Biotechnology and Biological Sciences Research Council (BBSRC) David Phillips Fellowship (BB/N020146/1). This work was funded by NERC (NE/S000771/1).

## Conflict of Interest

The authors declare that the research was conducted in the absence of any commercial or financial relationships that could be construed as a potential conflict of interest.

## Publisher’s Note

All claims expressed in this article are solely those of the authors and do not necessarily represent those of their affiliated organizations, or those of the publisher, the editors and the reviewers. Any product that may be evaluated in this article, or claim that may be made by its manufacturer, is not guaranteed or endorsed by the publisher.
