# Supplementary material for: Greater Phage Genotypic Diversity Constrains Arms-Race Coevolution
Source: Front Cell Infect Microbiol. 2022 Mar 4;12:834406. doi: 10.3389/fcimb.2022.834406 (PMC8931298; doi:10.3389/fcimb.2022.834406)
Supplement: Supplementary file 1 [file DataSheet_1.pdf]

Supplementary material: Greater phage genotypic diversity constrains arms-race coevolution

Authors

Meaghan Castledine[1], Pawel Sierocinski[1], Mhairi Inglis[1], Suzanne Kay[1], Alex Hayward[1], Angus Buckling[1], Daniel Padfield[1]

Corresponding author

Meaghan Castledine: mcastledine96@gmail.com

Contents:

Table S1. Multiple contrasts of bacterial resistance to their contemporary phage over time within each treatment.

Table S2. Means and standard errors of phage susceptible and resistant bacterial clones over time, within each treatment.

Table S3. Multiple contrasts of bacterial resistance to phage over time within each treatment.

Tables

Table S1. Multiple pairwise comparisons comparing the probability of a bacterial clone being susceptible to its contemporary phage through time (1, 2 and 3 corresponding to days 4, 8 and 12). Tests performed on the log odds ratio scale and p-values adjusted using the Tukey method of comparing a family of three estimates. Significant contrasts in bold. Phage 1, P1; Phage 2; P2. Values given to 3 significant figures.

Treatment	Time contrast	Odds-ratio	SE	z-ratio	p-value
**Phage 1**	**1 / 2**	**0.08660000000**	**0.0579000**	**-3.66000**	**0.00074700**
Phage 1	1 / 3	0.25500000000	0.1730000	-2.02000	0.10800000
Phage 1	2 / 3	2.94000000000	1.5100000	2.10000	0.08990000
Phage 2	1 / 2	0.00000000812	0.0000354	-0.00427	1.00000000
Phage 2	1 / 3	0.00000000571	0.0000249	-0.00435	1.00000000
Phage 2	2 / 3	0.70300000000	0.4320000	-0.57300	0.83500000
**Phage 1 and 2**	**1 / 2**	**0.06480000000**	**0.0538000**	**-3.30000**	**0.00282000**
**Phage 1 and 2**	**1 / 3**	**0.01870000000**	**0.0157000**	**-4.76000**	**0.00000583**
**Phage 1 and 2**	**2 / 3**	**0.28900000000**	**0.1240000**	**-2.90000**	**0.01030000**

Table S2. The proportion of phage susceptible and resistant bacterial populations when tested between three bacteria and phage time points (Tp) in each treatment. Values given to 3 significant figures.

Treatment	Bacteria Tp	Phage Tp	Susceptible	Resistant	SE
Phage 1	Day 4	Day 4	0.02780	0.972	0.01180
Phage 1	Day 4	Day 8	0.07640	0.924	0.06920
Phage 1	Day 4	Day 12	0.00694	0.993	0.00694
Phage 1	Day 8	Day 4	0.34000	0.660	0.11800
Phage 1	Day 8	Day 8	0.13900	0.861	0.07970
Phage 1	Day 8	Day 12	0.07640	0.924	0.06920
Phage 1	Day 12	Day 4	0.31200	0.688	0.10800
Phage 1	Day 12	Day 8	0.13900	0.861	0.08350
Phage 1	Day 12	Day 12	0.07640	0.924	0.06920
Phage 2	Day 4	Day 4	0.00000	1.000	0.00000
Phage 2	Day 4	Day 8	0.00000	1.000	0.00000
Phage 2	Day 4	Day 12	0.04170	0.958	0.04170
Phage 2	Day 8	Day 4	0.09720	0.903	0.04790
Phage 2	Day 8	Day 8	0.04170	0.958	0.03480
Phage 2	Day 8	Day 12	0.00000	1.000	0.00000
Phage 2	Day 12	Day 4	0.31200	0.688	0.11400
Phage 2	Day 12	Day 8	0.23600	0.764	0.09500
Phage 2	Day 12	Day 12	0.05560	0.944	0.04270
Phage 1 and 2	Day 4	Day 4	0.01390	0.986	0.01390
Phage 1 and 2	Day 4	Day 8	0.06250	0.938	0.04120
Phage 1 and 2	Day 4	Day 12	0.04860	0.951	0.02800
Phage 1 and 2	Day 8	Day 4	0.13900	0.861	0.05830
Phage 1 and 2	Day 8	Day 8	0.11800	0.882	0.06680
Phage 1 and 2	Day 8	Day 12	0.09720	0.903	0.06790
Phage 1 and 2	Day 12	Day 4	0.18100	0.819	0.08870
Phage 1 and 2	Day 12	Day 8	0.16000	0.840	0.07910
Phage 1 and 2	Day 12	Day 12	0.22200	0.778	0.09960

Table S3. Multiple pairwise comparisons comparing the probability of a bacterial clone being phage susceptible over time (1, 2 and 3 corresponding to days 4, 8 and 12) within each treatment. Tests performed on the log odds ratio scale and p-values adjusted using the Tukey method of comparing a family of three estimates. Significant contrasts in bold. Values given to 3 significant figures.

Treatment	Contrast	Odds-ratio	SE	z-ratio	p-value
**Phage 1**	**1 / 2**	**10.600**	**10.300**	**2.4500**	**0.037800**
**Phage 1**	**1 / 3**	**68.700**	**73.700**	**3.9400**	**0.000237**
Phage 1	2 / 3	6.460	7.160	1.6800	0.212000
Phage 2	1 / 2	2.680	2.720	0.9740	0.593000
**Phage 2**	**1 / 3**	**15.200**	**17.500**	**2.3700**	**0.047000**
Phage 2	2 / 3	5.680	6.680	1.4800	0.302000
Phage 1 and 2	1 / 2	0.950	0.899	-0.0547	0.998000
Phage 1 and 2	1 / 3	0.598	0.561	-0.5480	0.847000
Phage 1 and 2	2 / 3	0.630	0.590	-0.4940	0.874000
